# Trajectory of Post-COVID Self-Reported Fatigue and Dyspnoea in Individuals Who Had Been Hospitalized by COVID-19: The LONG-COVID-EXP Multicenter Study

**DOI:** 10.3390/biomedicines11071863

**Published:** 2023-06-29

**Authors:** César Fernández-de-las-Peñas, Ignacio Cancela-Cilleruelo, Jorge Rodríguez-Jiménez, Stella Fuensalida-Novo, José D. Martín-Guerrero, Oscar J. Pellicer-Valero, Ana I. de-la-Llave-Rincón

**Affiliations:** 1 Department of Physical Therapy, Occupational Therapy, Physical Medicine and Rehabilitation, Universidad Rey Juan Carlos, 28922 Madrid, Spain; ignacio.cancela@urjc.es (I.C.-C.); jorge.rodriguez@urjc.es (J.R.-J.); stella.fuensalida@urjc.es (S.F.-N.); anaidabel.delallave@urjc.es (A.I.d.-l.-L.-R.); 2 Intelligent Data Analysis Laboratory, Department of Electronic Engineering, ETSE (Engineering School), Universitat de València (UV), 46100 Valencia, Spain; jose.d.martin@uv.es; 3 Image Processing Laboratory (IPL), Universitat de València, Parc Científic, 46980 València, Spain; oscar.pellicer@uv.es

**Keywords:** COVID-19, fatigue, dyspnoea, symptoms, trajectory, Sankey plots

## Abstract

Fatigue and dyspnoea are common post-COVID symptoms. The aim of this study was to apply Sankey plots and exponential bar plots for visualizing the evolution and trajectory of post-COVID fatigue and dyspnoea symptoms in a cohort of previously hospitalized COVID-19 survivors. A total of 1266 previously hospitalized patients due to COVID-19 participated in this multicentre study. They were assessed at hospital admission (T0), 8.4 months (T1), 13.2 months (T2) and 18.3 months (T3) after hospital discharge and were asked about the presence of self-reported fatigue or dyspnoea symptoms. Fatigue was defined as a self-perceived feeling of constant tiredness and/or weakness whereas dyspnoea was defined as a self-perceived feeling of shortness of breath at rest. We specifically asked for fatigue and dyspnoea that participants attributed to the infection. Clinical/hospitalization data were collected from hospital medical records. The prevalence of post-COVID fatigue was 56.94% (*n* = 721) at T1, 52.31% (*n* = 662) at T2 and 42.66% (*n* = 540) at T3. The prevalence of dyspnoea at rest decreased from 28.71% (*n* = 363) at hospital admission (T0), to 21.29% (*n* = 270) at T1, to 13.96% (*n* = 177) at T2 and 12.04% (*n* = 153) at T3. The Sankey plots revealed that 469 (37.08%) and 153 (12.04%) patients exhibited fatigue and dyspnoea at all follow-up periods. The recovery exponential curves show a decreased prevalence trend, showing that fatigue and dyspnoea recover the following three years after hospitalization. The regression models revealed that the female sex and experiencing the symptoms (e.g., fatigue, dyspnoea) at T1 were factors associated with the presence of post-COVID fatigue or dyspnoea at T2 and T3. The use of Sankey plots shows a fluctuating evolution of post-COVID fatigue and dyspnoea during the first two years after infection. In addition, exponential bar plots revealed a decreased prevalence of these symptoms during the first years after. The female sex is a risk factor for the development of post-COVID fatigue and dyspnoea.

## 1. Introduction

Current knowledge supports that the severe acute respiratory syndrome coronavirus 2 (SARS-CoV-2), the agent causing the coronavirus disease, 2019 (COVID-19), affects multiple systems and organs, however, it seems that the respiratory system is probably the most affected [1]. Fever, cough, and dyspnoea are the symptoms most commonly experienced at COVID-19 onset [2]. Although neither fatigue nor dyspnoea are exclusive of COVID-19 [3]; the presence of dyspnoea as an onset SARS-CoV-2 associated-symptom has been found to be associated with COVID-19 severity [4].

Fatigue and dyspnoea are also prevalent symptoms experienced in post-acute phase of COVID-19 disease [5]. Among the different terms used for defining the potential presence of post-COVID symptoms, long-COVID [6] or post-COVID-19 conditions [7] are the most accepted in the scientific literature. A Delphi study proposed the following definition for post-COVID-19 condition: “Post-COVID-19 condition occurs in people with a history of probable/confirmed SARS-CoV-2 infection, usually three months from the onset of COVID-19 with symptoms that last for at least two months and cannot be explained by an alternative medical diagnosis” [7]. More than 100 post-COVID symptoms have been described being fatigue and dyspnoea the most prevalent [8]. Meta-analyses specifically investigating post-COVID fatigue have reported pooled prevalence rates ranging from 32% (68 studies; *n* = 25,268 subjects) [9] to 42% (41 studies, *n* = 9362 subjects) [10] during the first six months after the infection. The presence of post-COVID fatigue is associated with worse health-related quality of life [11], and it is probably one of the most bothersome post-COVID symptoms in addition to dyspnoea. In fact, the prevalence of post-COVID dyspnoea (102 studies, *n* = 42,872 patients) has been estimated to be around 26% when is self-reported and up to 41% when using a specific dyspnoea scale, e.g., the Medical Research Council (MRC scale) [12]. The Global Burden of Disease Long COVID study (*n* = 1.2 million individuals with symptomatic SARS-CoV-2 infection) concluded that 51.0% of COVID-19 survivors self-reported persistent post-COVID fatigue and bodily pain (cluster one) whereas 60.4% reported respiratory problems (cluster two) during the first months after the acute infection [13]. In addition, up to 15.1% of subjects still experienced symptoms 12 months after the infection [13].

Most studies investigating the presence of post-COVID fatigue and dyspnoea have used cross-sectional design assessing the presence of these symptoms just once and also had commonly used follow-up periods no longer than one year after COVID-19 [8,9,10,11,12,13]. The LONG-COVID-EXPERIENCE study is a multicentre cohort study investigating the evolution of 2000 previously hospitalized COVID-19 survivors during the first year after hospitalization. This study analysed the evolution of post-COVID fatigue and dyspnoea up to the first year after hospitalization [14]. Understanding the longitudinal trajectory of post-COVID fatigue and dyspnoea symptoms could have potential implications for optimising patient treatment [15] and public health outcomes [16]. The current paper shows the complete follow-up analysis of the LONG-COVID-EXPERIENCE study on the evolution of fatigue and dyspnoea from the onset of the disease, up to 6, 12 and 18 months after hospital discharge. Thus, this report presents the application of a novel method, e.g., Sankey plots, for visualization of the evolution of these particular post-COVID symptoms. In addition, we also applied again exponential bar plot analyses for predicting the exponential trajectory of post-COVID fatigue and dyspnoea.

## 2. Methods

### 2.1. Participants

The LONG-COVID-EXP-CM is a multicentre study including a cohort of individuals who had been hospitalized during the first wave of the pandemic (from 10 March to 31 May 2020) in five hospitals of Madrid (Spain) due to SARS-CoV-2 (ICD-10 code). All patients should have been diagnosed of COVID-19 at hospital admission by real-time reverse transcription-polymerase chain reaction (RT-PCR) assay of nasopharyngeal/oral swab samples. As originally designed in the study, a randomized sample of 400 subjects from each participating hospital was selected from all of the individuals who had been hospitalized during the first wave of the pandemic in these hospitals (*n* = 7150). The Local Ethics Committee of all hospitals approved the study (HUFA20/126, HUF/EC1517, HUIL/092-20, HCSC20/495E, and HSO25112020). Verbal informed consent was obtained from all the participants before collecting any data.

### 2.2. Procedure

The procedure of this multicentre cohort study has been previously described [14]. Briefly, clinical and hospitalization data were collected from hospital medical records. Participants were scheduled for a telephone semi-structured interview conducted by trained healthcare professionals at six, twelve and eighteen months after hospitalization. Participants were systematically asked about the presence of self-reported fatigue and dyspnoea symptoms. Fatigue was defined as a self-perceived feeling of constant tiredness or weakness. Dyspnoea was defined as self-perceived feeling of shortness of breath at rest. We specifically asked for fatigue and dyspnoea symptoms that subjects attributed to COVID-19. Particularly, at the first follow-up, we focused on symptoms starting no later than three months after infection or hospital discharge [7]. Finally, medical records were revised to identify if patients self-reporting post-COVID fatigue and/or dyspnoea have been diagnosed of any medical condition explaining these symptoms.

### 2.3. Sankey Plots

Sankey plots are flow diagrams used for visualization of the flow of quantitative data permitting assessment of the evolution of patients over time [17]. The X axis represents each time-point (onset, six, twelve or eighteen months after infection), while the Y axis represents the percentage of individuals suffering (or not) from each symptom (fatigue and dyspnoea). The state of the individuals at that particular time-point is represented in the Sankey plot as darker vertical bars. The flow (or change) of the individuals between states (positive or negative in a symptom) is depicted by arcs with a different width according and proportional to the percentage (from the total sample) of subjects. The percentage of subjects with or without each particular symptom is placed into the right side of the vertical bars whereas the percentage of individuals flowing from positive to negative (or the opposite) of each symptom is annotated into the left side of the vertical bar [17].

### 2.4. Exponential Bar Plots

The Matplotlib 3.3.4 software was used to create exponential curves. In the current study, exponential curves were fitted according to the formula $y=Ke^{ct}$, where y represents the point prevalence of each symptom (i.e., fatigue or dyspnoea) at a specific time moment t (in months), and K and c are the parameters of the model.

### 2.5. Statistical Analysis

Additionally, demographic features (e.g., age, weight and height), medical comorbidities and all variables collected at hospital admission (COVID-19 onset, T0) and at the first follow-up (T1) were entered into multivariate logistic regression models to identify those variables associated with the development of post-COVID fatigue or dyspnoea at T2 and T3 by using Python’s library statsmodels 0.11.1. Adjusted odds ratio (OR) with their confidence intervals (95%CI) are presented. The level of significance was set a priori at 0.05.

## 3. Results

From a sample of 2000 individuals hospitalized due to SARS-CoV-2 randomly selected to participate, a total of 1969 (46.5% women, age: 61, SD: 16 years old) were included at baseline (T0) and 6 months (T1); 1593 were assessed at 12 months (T2) and 1266 were evaluated at 18 months (T3). Thus, analyses were conducted on the sample (*n* = 1266, 64.3% from the total) completing all follow-up periods: T1 (mean: 8.4, range 6 to 10), T2 (mean: 13.2, range 11 to 15) and T3 (mean: 18.3, range 16 to 21) months after hospitalization. Table 1 shows COVID-19-associated symptoms at hospital admission and medical comorbidities of the sample.

**Figure 1:**
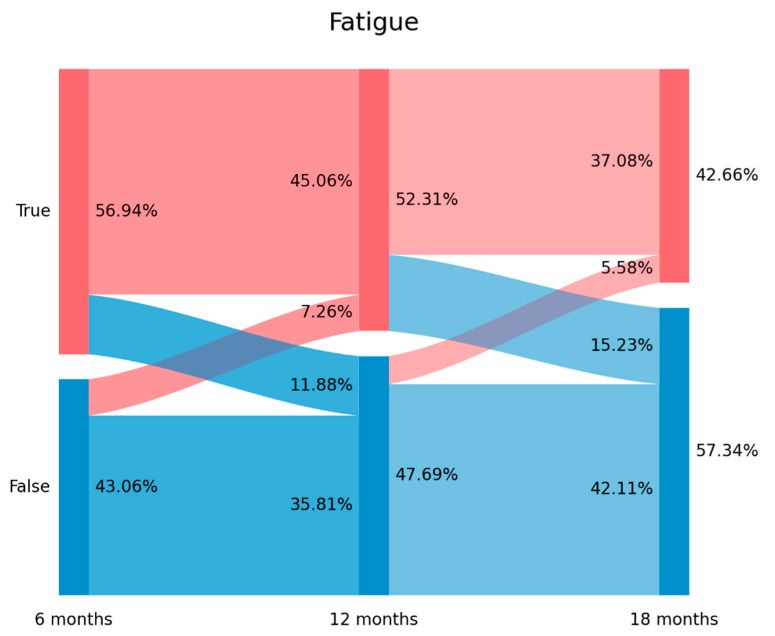
Visualization of post-COVID fatigue from T1 (8.4 months after hospitalization) follow-up period (left side) to T3 (18.3 months after hospital discharge) follow-up period with a Sankey plot.

The prevalence of post-COVID fatigue was 56.94% (*n* = 721) at T1, 52.31% (*n* = 662) at T2, and 42.66% (*n* = 540) at T3. Figure 1 graphs the Sankey plots of post-COVID fatigue. Looking at Figure 1, 20.8% of subjects (*n* = 150/721) experiencing fatigue at T1 did not report fatigue at T2 (11.88% arc from true at T1 to false at T2). Interestingly, 16.9% (*n* = 92/545) of subjects not experiencing fatigue at T1 self-reported fatigue at T2 (7.26% arc from false at T1 to true at T2) Finally, 29.1% (*n* = 193/662) of subjects with fatigue at T2 did not report this symptom at T3 (15.23% arc from true at T2 to false at T3). The plot revealed that 469 patients (37.08% of the sample) exhibited post-COVID fatigue during all the follow-up periods.

**Figure 2:**
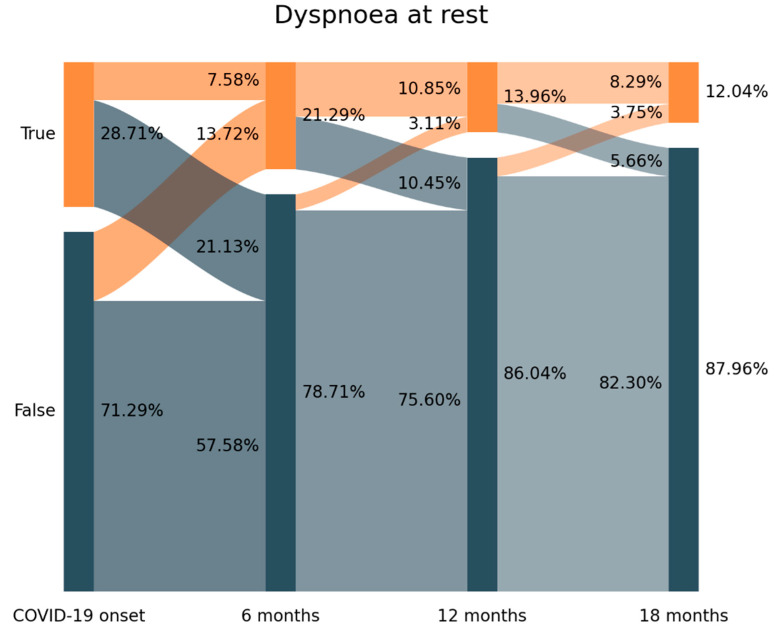
Visualization of the evolution of dyspnoea from T0 (hospital admission, COVID-19 onset) on the left side) to T3 (18.3 months after hospital discharge) follow-up period with a Sankey plot.

The prevalence of dyspnoea at rest decreased from 28.71% (*n* = 363) at hospital admission (T0), to 21.29% (*n* = 270) at T1, to 13.96% (*n* = 177) at T2 and 12.04% (*n* = 153) at T3 (Figure 2). Looking at Figure 2, 73.8% of those subjects (*n* = 268/363) experiencing dyspnoea at hospital admission (T0) had recovered at T1 (21.13% arc from true at T0 to false at T1).

Further, 64.4% (*n* = 174/270) of individuals reporting dyspnoea at rest at T1 developed “new-onset” post-COVID dyspnoea since they did not experience dyspnoea at T0 (13.72% arc from false at T0 to true at T1). A similar tendency was seen between T1-T2 and T2-T3 but with a small number of individuals. The Sankey plot revealed that 153 patients (12.04% of the sample) self-reported dyspnoea at rest as a symptom of the acute infection and throughout all the follow-up periods.

**Figure 3:**
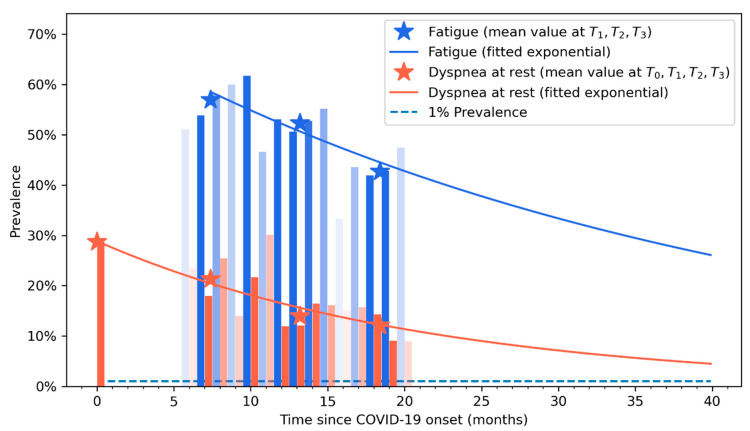
Exponential recovery curve of self-reported fatigue (in blue) and dyspnoea (in orange) symptoms. Vertical bars represent the percentage of patients that self-reported fatigue (in blue) or dyspnoea (light red) at each follow-up period. Asterisks represents the prevalence point of each symptom at each follow-up (T0, T1, T2, and T3).

Figure 3 graphs the fitted exponential curves visualizing a decreased prevalence trend in both post-COVID fatigue and dyspnoea during the following three years after the infection. From this figure, it can be observed that natural tendency of post-COVID fatigue and dyspnoea is to recover within the subsequent months after the infection; however, it can be also observed that fatigue symptoms could be present at least five or six years after the acute infection (based on this predictive model).

The regression models revealed that the female sex and experiencing the particular symptom at the first follow-up were factors significantly associated with developing post-COVID fatigue and dyspnoea: fatigue at 12 months (female sex: OR 1.464, 95%CI 1.077–2.128, *p* = 0.046; fatigue at 6 months: OR 3.978, 95%CI 2.571–6.154, *p* < 0.001), fatigue at 18 months (female sex: OR 1.464, 95%CI 1.055–2.032, *p* = 0.022; fatigue at 6 months: OR 2.010, 95%CI 1.331–3.035, *p* = 0.001), dyspnoea at rest at 12 months (female sex: OR 1.953, 95%CI 1.140–3.347, *p* = 0.015; dyspnoea at rest at 6 months: OR 7.062, 95%CI 3.828–13.027, *p* < 0.001), dyspnoea at rest at 18 months (dyspnoea at rest at 6 months: OR 6.867, 95%CI 3.477–13.563, *p* < 0.001). No other association was identified.

## 4. Discussion

As far as the author’s knowledge, this is the first study using two visualization approaches (e.g., Sankey plots and exponential curves) for analysing the recovery of post-COVID fatigue and dyspnoea in a cohort of individuals who had been previously hospitalized due to SARS-CoV-2. The Sankey plots revealed a fluctuating evolution of post-COVID fatigue and dyspnoea at rest during the first year after the acute infection. Additionally, the exponential bar plots revealed a progressive decrease in the prevalence of post-COVID fatigue and dyspnoea during the first three years after the infection.

Previous meta-analyses including cross-sectional studies had reported an overall prevalence of post-COVID fatigue ranging from 32% to 42% during the first six months after infection [9,10]. Another meta-analysis observed similar prevalence rates of post-COVID fatigue between hospitalized (28.4%, 95%CI 24.7–32.5%) and non-hospitalized (34.8%, 95%CI 17.6–57.2%) subjects during the first six months after infection [18]. Yang et al. reported an overall prevalence of post-COVID fatigue of 26.2% one year after the infection [19]. Our study showed prevalence rates of post-COVID fatigue ranging from 56.9% to 42.6%, data slightly higher than the current literature [9,10]. Differences in study designs (cross-sectional vs. longitudinal), differences in follow-ups (one-six months after infection), population (hospitalized vs. non-hospitalized) and collection procedure (self-reported, phone interview, face-to-face) can explain the heterogeneous prevalence rates among studies. The exponential curves graph that the prevalence of post-COVID fatigue decreased with time, hence, longer follow-up periods from the SARS-CoV-2 infection leads to lower rates, in agreement with current literature [13]. However, the exponential graph suggests that post-COVID fatigue and dyspnoea symptoms can last up to five years after infection. It should be noted that fatigue was not specifically assessed at hospital admission, since fever, cough and dyspnoea are symptoms most commonly experienced at the acute phase of SARS-CoV-2 infection [2].

Dyspnoea at rest is probably one of the most bothersome COVID-19 associated symptoms. We observed a prevalence of 28.7% at hospital admission in agreement with current data [2]. We also reported a prevalence of self-reported post-COVID dyspnoea from 12% to 21.3%, data also similar to current meta-analyses pooling prevalence rates between 15.5% and 26% [12,19]. Again, the exponential recovery curves graph that the prevalence of post-COVID dyspnoea decreased with time [13] but it can be present up to 3–4 years after hospitalization.

One of the most interesting findings is the use of Sankey plots which permitted to identify a fluctuating nature of post-COVID symptoms, e.g., fatigue and dyspnoea, as previously suggested [20]. In fact, Fernández-de-las-Peñas et al. proposed the terms “new-onset” and “persistent” post-COVID symptom according to the presence of a symptom at the acute phase of infection or just at the post-COVID phase [21]. Analysing the Sankey plots, we were able to identify:

1. new-onset post-COVID dyspnoea: subjects experiencing dyspnoea after the infection but not at the acute phase (13.72% arc from false at T0 to true at T1 on Figure 2)
2. persistent post-COVID dyspnoea: patients suffering from dyspnoea from the onset of the acute infection and throughout all the follow-up periods (12.04% of the sample in Figure 2)

Both new-onset and persistent post-COVID symptoms can be easily attributable to COVID-19 if the symptom started no later than three months after the acute infection [9]; nevertheless, we observed the presence of “delayed onset post-COVID symptoms”: individuals reporting post-COVID fatigue (7.26% arc from false at T1 to true at T2 at Figure 1) or post-COVID dyspnoea (3.11% from false at T1 to true at T2 at Figure 2) at a longer follow-up period in relation to the acute phase of the infection. This third type of post-COVID symptom is more difficult to attribute to SARS-CoV-2 since it appeared several months after the infection. It is possible that other factors (e.g., post-traumatic stress, medical comorbidities, and reinfections) are more related to the development of this “delayed” post-COVID symptom rather than just the acute initial infection.

Multiple mechanisms, e.g., including deconditioning, restrictive/obstructive airflow limitation or systemic inflammation have been proposed for explaining the presence of fatigue and dyspnoea after the acute infection, but the body of evidence is inconclusive. In fact, the presence of lung damage, e.g., fibrosis, would lead to a post-COVID sequelae and not to a post-COVID symptom which is present in the absence of any medical condition able to explain the symptoms [9]. Damage in lung function is probably the main hypothesis proposed for the presence of post-COVID fatigue and dyspnoea since a persistent reduction in diffusing capacity of the lungs for carbon monoxide has been found in almost 40% of COVID-19 survivors up to one year after the infection [22]. In addition, up to 60% of COVID-19 survivors exhibit at least one chest computed tomography (CT) abnormality between three to six months after infection, which would be a post-COVID sequalae [23]. The presence of abnormal lung function could be associated with a lower percentage of predicted oxygen uptake at peak exercise (%VO2pred) as a potential explanation of the impaired exercise tolerance observed in patients with long COVID [24]. It is, therefore, possible that, in some people with prior sub-optimal mitochondrial function, the SARS-CoV-2 can tip the host into a chronic inflammatory cycle inducing platelet dysfunction. In fact, long-COVID is described as a virally induced chronic and self-perpetuating metabolically imbalanced non-resolving state characterized by mitochondrial dysfunction where reactive oxygen species continually drive inflammation [25]. It has been recently found that subjects with post-COVID symptoms have a decrease in the expression of regulatory T cells (Tregs) which could increase the chance of respiratory failure [26]. This generalised state could explain why 45.2% of COVID-19 fulfil chronic fatigue syndrome criteria (52 studies; *n* = 127,117 patients) in the following months after the infection [27]. In the current study, medical history was used to identify if these individuals have been diagnosed with any medical condition, e.g., lung damage or fibrosis, explaining the presence of post-COVID fatigue or dyspnoea, since the presence of a medical condition would lead to a post-COVID sequalae and these symptoms could not be considered as post-COVID-19 condition. Nevertheless, the lack of a medical condition does not exclude the potential presence of abnormal lung function, which was not assessed in the current study.

Finally, we identified that female sex and experiencing the symptom, e.g., fatigue or dyspnoea, at the first follow-up period were risk factors associated with post-COVID fatigue and dyspnoea at longer follow-up periods. A previous meta-analysis concluded that post-COVID fatigue and dyspnoea were associated with severe/critical COVID-19 disease, hospitalization and female sex [12]. The fact that female sex is a risk factor for long-COVID is well supported in the former literature [28]. Second, since we only included a cohort of hospitalized survivors, factors of hospitalization and severe/critical COVID-19 could not be assessed in the current study. In fact, although single studies suggest no association between COVID-19 severity and the development of overall post-COVID symptoms, the meta-analysis Dirican and Bal showed an association between the severity of COVID-19 disease and post-COVID dyspnoea or fatigue [29]. Fatigue and dyspnoea are respiratory symptoms, and since SARS-CoV-2 mainly targets/attacks the respiratory system, the association between COVID-19 severity with these symptoms, and no other symptoms related to other organs, would be expected. Since we included subjects with severe COVID-19 who need hospitalization during the first wave of the pandemic, we were unable to identify this association. In addition, ICU admission, i.e., subjects with critical COVID-19 were not either associated with the development of post-COVID fatigue and dyspnoea.

Although this cohort study used two novel methods of visualization and analysis of post-COVID fatigue and dyspnoea, potential weaknesses are recognized. First, the study only included previously hospitalized COVID-19 survivors. We do not know if similar data will be obtained in a cohort of non-hospitalized individuals. Second, we collected data by telephonic interview, a procedure which could has a potential bias. However, it is important to note that the use of telephonic interviews is the only way to assess large cohorts like that one included in the current study (over 1000 of patients during long-term follow-up periods). Third, fatigue and dyspnoea were self-reported. It is possible that the use of specific questionnaires could lead to different results. It is interesting that the prevalence of post-COVID dyspnoea was higher (41%) when a specific scale was used when compared with the prevalence (26%) of self-reported symptomatology [12]. Finally, it should be considered that the decrease prevalence identified in the current study could be associated with other factors not considered, such as vaccine status or potential treatments received by the patients during the follow-up. Although we asked for specific treatments received by the patients, most of them answered that they have not been treated during the follow-up period. In relation to vaccine status, although COVID-19 vaccines can decrease the risk of developing of post-COVID-19 condition if they are administered before the infection, their effects on individuals with current post-COVID symptoms are not clear which most meta-analysis reported that mixed effects on current long-COVID [30,31].

## 5. Conclusions

The use of Sankey plots revealed a fluctuating evolution of post-COVID fatigue and dyspnoea at rest during the first years after infection in a cohort of previously hospitalized COVID-19 survivors. Additionally, exponential bar plots also visualized a decrease in these symptoms the first three-four years after the acute infection. Female sex was a risk factor associated with the perpetuation of these symptoms at longer follow-ups. In addition, experiencing post-COVID fatigue and dyspnoea soon after the infection was also a risk factor associated with experiencing these symptoms at longer follow-ups.

## Figures and Tables

**Table 1:** Demographic and clinical data of the sample (*n* = 1266).

Age, mean (SD), years	61 (16.5)
Female (%)	578 (45.6%)
Weight, mean (SD), kg	74.5 (14.5)
Height, mean (SD), cm.	165 (19.0)
Main Symptoms at hospital admission, n (%)—T0	
Fever	948 (74.9%)
Myalgia	374 (29.5%)
Dyspnoea	361 (28.5%)
Cough	360 (28.4%)
Headache	135 (16.7%)
Diarrhoea	105 (8.3%)
Anosmia	105 (8.3%)
Ageusia	66 (7.0%)
Throat Pain	66 (5.2%)
Vomiting	39 (3.0%)
Medical co-morbidities	
Hypertension	336 (26.5%)
Other (Cancer, Kidney Disease)	207 (16.3%)
Diabetes	158 (12.5%)
Cardiovascular Disease	141 (11.2%)
Asthma	85 (6.7%)
Obesity	57 (4.5%)
Chronic Obstructive Pulmonary Disease	47 (3.7%)
Rheumatological Disease	16 (1.3%)
Stay at the hospital, mean (SD), days	10.5 (10.8)
Intensive Care Unit (ICU) admission	78 (6.2%)

## Data Availability

All data are presented in the text.
